# Discovery of METTL3 Small Molecule Inhibitors by Virtual Screening of Natural Products

**DOI:** 10.3389/fphar.2022.878135

**Published:** 2022-04-27

**Authors:** Yue Du, Yongliang Yuan, Le Xu, Fang Zhao, Wenbin Wang, Yiping Xu, Xin Tian

**Affiliations:** ^1^ Department of Pharmacy, The First Affiliated Hospital of Zhengzhou University, Zhengzhou, China; ^2^ Henan Key Laboratory of Precision Clinical Pharmacy, The First Affiliated Hospital of Zhengzhou University, Zhengzhou, China; ^3^ Departments of Urology, The First Affiliated Hospital of Zhengzhou University, Zhengzhou, China

**Keywords:** METTL3, m^6^A, inhibitor, natural products, cancer

## Abstract

N^6^-Methyladenosine (m^6^A) is the most prevalent mRNA modification in mammalian cells that is mainly catalyzed by the methyltransferase complex of methyltransferase-like 3 and methyltransferase-like 14 (METTL3-METTL14). Many lines of evidence suggest that METTL3 plays important roles in several diseases such as cancers and viral infection. In the present study, 1,042 natural products from commercially available sources were chosen to establish a screening library, and docking-based high-throughput screening was performed to discover potential METTL3 inhibitors. The selected compounds were then further validated by an *in vitro* methyltransferase inhibition assay in which m^6^A content was determined by LC-MS/MS. A cellular assay of the inhibition of m^6^A methylation was performed to determine the METTL3 inhibitory activity of the selected compound. CCK-8 assay was applied to evaluate the effects of the selected compound on tumor cell viability. Additionally, binding mode analysis, molecular dynamics (MD) simulation, and binding free energy analysis were performed to study the process and characteristics of inhibitor binding. Finally, quercetin was identified as a METTL3 inhibitor with an IC_50_ value of 2.73 μM. The cellular assay of m^6^A methylation inhibition showed that quercetin decreased m^6^A level in a dose-dependent manner in MIA PaCa-2 pancreatic cancer cells. CCK-8 assay showed quercetin efficiently inhibited the proliferation of MIA PaCa-2 and Huh7 tumor cells, with IC_50_ values 73.51 ± 11.22 μM and 99.97 ± 7.03 μM, respectively. Molecular docking studies revealed that quercetin filled the pocket of the adenosine moiety of SAM but not the pocket of the SAM methionine in the METTL3 protein, and hydrogen bonds, hydrophobic interactions, and pi-stacking were formed. The values of the root mean square deviation (RMSD), the root mean square fluctuations (RMSF), and binding free energy suggested that quercetin can efficiently bind to the pocket of the METTL3 protein and form a stable protein-ligand complex. The present study is the first to identify METTL3 inhibitors from natural products, thus providing a basis for subsequent research and facilitating the development of METTL3-targeting drugs for diseases.

## Introduction

To date, more than 100 types of chemical modifications have been identified in cellular RNAs, and these modifications play essential roles in a wide range of cellular processes. N^6^-Methyladenosine (m^6^A) is the most abundant internal mRNA modification in eukaryotic cells (Roundtree et al., 2017). By influencing several aspects of mRNA metabolism, such as pre-mRNA processing (Liu et al., 2015), translation (Wang et al., 2015), nuclear export (Edens et al., 2019), and decay (Shi et al., 2017), the epigenetic modification of m^6^A has functional roles in various processes such as cell differentiation (Geula et al., 2015), embryonic development (Ping et al., 2014), and heat stress response (Zhou et al., 2015). The dynamics and reversibility of m^6^A modification are regulated by three distinct classes of protein factors, including “readers” (m^6^A-binding proteins), “writers” (m^6^A methyltransferases), and “erasers” (m^6^A demethylases). The writers and erasers install and remove m^6^A modification, respectively, and the readers recognize it (Zaccara et al., 2019). The most important RNA methyltransferases comprise the heterodimer complex formed by methyltransferase-like 3 (METTL3) and methyltransferase-like 14 (METTL14). METTL3 primarily functions as the catalytic core, while METTL14 facilitates RNA substrate binding and stabilizes the complex (Wang et al., 2016; Śledź and Jinek, 2016). This complex is regulated by a regulatory subunit known as Wilms’ tumor 1-associating protein (WTAP) (Liu et al., 2014). The METTL3-METTL14 complex catalyzes a methyl group in S-adenosyl-L-methionine (SAM) transfer to N^6^ atom of adenine in RNA consensus sequence (Wang et al., 2016). The dysregulation of the process of m^6^A modification could lead to disease development.

As the key catalytic subunit of methyltransferase, METTL3 has a wide range of biological functions but remains elusive. As reported earlier, METTL3 plays diverse roles in cancers; in most cases, it acts as an oncogene to promote the initiation and development of a variety of cancers, such as promotion of translation and RNA stabilization to regulate cell proliferation, migration, invasion, and chemoresistance (Zeng et al., 2020). Moreover, many lines of evidence suggest that METTL3 plays important roles in various diseases such as cardiovascular diseases (Zhang et al., 2021), diabetes (Li et al., 2020), and viral infection (Hao et al., 2019). In an *in vivo* therapeutic study, the METTL3 inhibitor STM2457 was shown to treat myeloid leukemia (Yankova et al., 2021). Consequently, METTL3 is predicted to be an attractive therapeutic target for treating diseases, including cancers. Additionally, small molecule inhibitors of METTL3 are helpful to elucidate the roles and regulation mechanisms of METTL3 in health and disease.

The disclosure of the METTL3-METTL14 cocrystals has provided structural information for designing METTL3 inhibitors (Wang et al., 2016). To date, UZH1a, UZH2, STM2457, and 43n have been reported as METTL3 inhibitors in previous studies (Dolbois et al., 2021; Moroz-Omori et al., 2021; Yankova et al., 2021; Lee et al., 2022); however, none of them have undergone clinical studies. Therefore, there is an urgent need to search for new potential candidate inhibitors to enrich the structural diversity of METTL3 inhibitors. The complex molecular frameworks of natural products provide a series of unknown chemical types for discovering compounds with diverse biological activities (Rodrigues et al., 2016; Cheng et al., 2019). Many new drugs have been obtained from natural products and compounds derived from natural products (Li and Vederas, 2009). At present, many natural products (e.g., vincristine, taxol, and doxorubicin) are used to treat a variety of malignancies (Da Rocha et al., 2001). However, despite being a huge resource no natural products have been reported or screened as METTL3 inhibitors.

**FIGURE 1:**
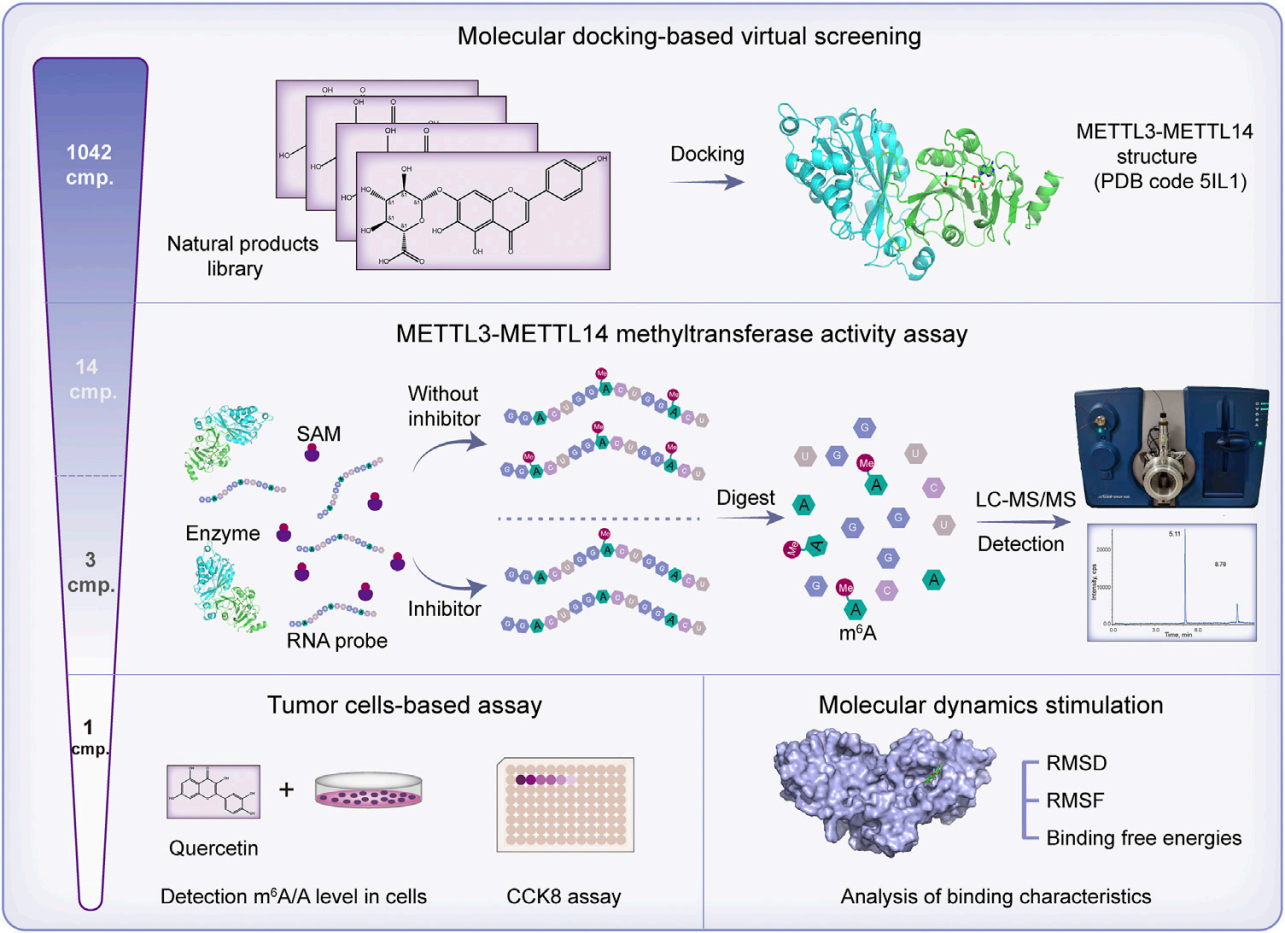
Schematic diagram of the identification of novel METTL3 inhibitor.

In the present study, the molecular docking method was carried out to virtually screen a library of 1,042 natural compounds based on the METTL3 crystal structure. On the basis of the virtual screening scores and commercially available sources, 14 compounds were selected to further evaluate their biological activity. After initial biological evaluation, three compounds were chosen to determine their IC_50s_ value against METTL3. Finally, quercetin was found to be the most potent METTL3 inhibitor. Next, the effect of quercetin on the m^6^A level and viability of tumor cells was evaluated. Moreover, binding mode analysis, molecular dynamics (MD) simulation, and binding free energy analysis were performed to study the binding characteristics between quercetin and METTL3 protein (Figure 1).

## Materials and Methods

### Chemicals and Reagents

Quercetin, scutellarin, cynaroside, naringin, hesperidin, diosmetin, luteolin, hesperitin, apigenin, naringenin, rutin, wogonin, and sophoricoside were purchased from Chengdu Must Bio-Technology Co., Ltd. (Chengdu, China). Baicalein was purchased from Solarbio (Beijing, China). The purity of all the natural products was ≥98%. The reference standard of adenosine (purity >99%) was purchased from Tokyo Chemical Industry (Shanghai, China). M^6^A (purity 97%) was purchased from J&K Chemical (Shanghai, China).

### Molecular Docking-Based Virtual Screening

For virtual screening of METTL3 inhibitors, approximately 1,042 natural products from commercially available sources were chosen to establish a screening library. The structural formula (SDF format) of the compounds was downloaded from the PubChem database and converted into PDB format. The crystal structure of the METTL3-METTL14 complex (PDB code: 5IL1) (Wang et al., 2016) with a resolution of 1.71 Å was downloaded from the Protein Data Bank (PDB, http://www.rcsb.org/pdb). Hydrogen atoms were added, and crystallographic water molecules were deleted. SAM and the METTL3-METTL14 complex were separated by AutoDock Vina 1.1.2. The library compound file in PDB format was imported into AutoDock Vina. Atomic charges were added, atomic types were assigned and all flexible bonds were rotatable by default. The library compounds were then docked into the SAM binding site of METTL3 by AutoDock Vina 1.1.2 (Trott and Olson, 2010). Compounds with affinity (kcal/mol) (free energy ≤ −7 kcal/mol) and readily available commercial sources were selected for biological evaluation using the METTL3 methyltransferase *in vitro* activity assay.

### Methyltransferase Activity Assay

M^6^A formation assay was performed to determine the methyltransferase activity of the METTL3-METTL14 complex. The sequence of the RNA probe was 5′-GGA​CUG​GAC​UGG​ACU​GGA​CU-3′ and was synthesized by Tsingke (Beijing, China). The METTL3-METTL14 protein and METTL3-METTL14 mutant protein were kindly provided by Ping Yin, professor at the National Key Laboratory of Crop Genetic Improvement and National Centre of Plant Gene Research, Huazhong Agricultural University, Wuhan, China. The methyltransferase reaction system was based on a previous report with some modifications (Wang et al., 2016). The 30 ul reaction mixture contained 15 mM HEPES pH 7.3, 50 mM KCl, 50 mM NaCl, 1 mM MgCl_2_, 1 mM dithiothreitol, 4% glycerol, 10 μM SAM, 2 μM RNA oligo, and 40 nM protein. The mixture was incubated at 30°C for 2 h. Next, proteinase K was added to each reaction mixture for digestion at 55°C for 60 min, and the reaction was stopped at 95°C for 10 min. S1 nuclease (Takara) and alkaline phosphatase (calf intestine, Takara) were added to the digested RNA to form mononucleosides, and the samples were vortexed at 37°C for 6 h. Subsequently, the samples were transferred to ultrafiltration tubes (molecular weight cutoff of 3 kDa) and centrifuged at 4°C, 14,000 g for 15 min. The liquid chromatography-tandem mass spectrometry (LC-MS/MS) method was applied to determine the m^6^A content by using a QTRAP 4500 mass spectrometer (AB Sciex, United States) according to the reported method (Liu R. et al., 2019).

The tested compounds that inhibited METTL3 activity could decrease m^6^A formation and thus reduce the m^6^A/A ratio. Percent inhibition was normalized to control wells without enzyme and without inhibition (DMSO alone). The percent inhibition of serial dilutions of compounds was calculated by measuring METTL3 activity. The half-maximal inhibitory concentration (IC_50_ value) of the compounds was calculated by measuring METTL3 activity over a dilution series of inhibitor concentrations. The response curves were generated using nonlinear regression “log (inhibitor) vs. response —variable slope (four parameters)” in GraphPad Prism 8.4.0.

### Cell Lines and Culture Conditions

The human pancreatic carcinoma cell line MIA PaCa-2 was purchased from the American Type Culture Collection (ATCC), and the human liver cancer cell line Huh7 was purchased from the National Collection of Authenticated Cell Cultures. All cells were grown in Dulbecco’s modified Eagle’s medium-high glucose (Sigma), supplemented with 10% v/v fetal bovine serum (Gibco, Life Technologies). The cells were cultured at 37°C in an incubator with 5% CO_2_.

### Quantification of the m^6^A/A Ratio in mRNA

METTL3 knockdown in MIA PaCa-2 cells was performed by transient transfection of siRNA. METTL3 siRNA and nonspecific siRNA oligonucleotides (siNC) were synthesized by RiboBio. The METTL3 siRNA sequence corresponded to the coding region (5′-CAG​TGG​ATC​TGT​TGT​GAT​A-3′) of the METTL3 gene. SiMETTL3 and siNC were transfected into MIA PaCa-2 cells using Lipofectamine RNAiMAX (Invitrogen) according to the manufacturer’s protocol. Quercetin was added to the cell culture at different concentrations. DMSO alone was used as a negative control. After 24 h incubation, the cells were washed with PBS and harvested. TRIzol reagent (Thermo Fisher) was used to extract total RNA according to the standard protocol. mRNA was isolated using VAHTSTM mRNA Capture Beads (Vazyme Biotech) according to the manufacturer’s instructions. S1 nuclease (Takara) and alkaline phosphatase (calf intestine, Takara) were used to digest the mRNA into mononucleotides. The LC-MS/MS method was applied to determine the m^6^A content as described above. The ratio of m^6^A/A was calculated on the basis of the detected concentrations.

### Cell Viability Assay

To evaluate the antitumor potential of quercetin, the viability of MIA PaCa-2 and Huh7 cells after treatment with quercetin was assessed using the Cell Counting Kit-8 (CCK-8) assay. Cells (2,500 cells per well) were seeded in 96-well plates, and after 24 h they were treated with different concentrations of quercetin. After 72 h of culture, the CCK-8 reagent was added to each well according to the instructions provided by the manufacturer of the CCK-8 Kit (Vazyme Biotech Co., Ltd.). The absorbance was measured at 450 nm using a microplate reader (MULTISKAN FC, Thermo Fisher Scientific). Cells treated with DMSO served as the control. The IC_50_ values were calculated by GraphPad Prism 8.4.0.

### MD and Binding Free Energy Calculation

MD simulation of the METTL3-METTL14/ligand complex was performed with Amber20 (Case et al., 2005; Salomon-Ferrer et al., 2013). The simulation was run using the ff14SB force field for METTL3-METTL14 protein and the general Amber force field (GAFF) for the ligands (Maier et al., 2015). The software package Gaussian 09 based on B3LYP/6-31G* was used to optimize the geometry of each ligand and to calculate electrostatic potential of the ligands (Frisch et al., 2009). Partial charges of the ligands were generated by the antechamber module for electrostatic potential fitting using the restrained electrostatic potential (RESP) method. The sander program in Amber20 was used for molecular mechanics optimization and MD simulation of the complex. The system was solvated using the TIP3P water box with a three-dimensional space of 10 Å × 10 Å × 10 Å, and counterions were added to maintain system neutrality. Before MD simulation, a two-step energy optimization process was performed on the system. Energy minimization was first conducted with 5,000 steps for the fixed solute molecular water, including the steepest descent method of 2,500 steps and the conjugate gradient method of 2,500 steps. Next, the energy minimization of 5,000 steps for the entire system was performed by releasing the constraints, which also include 2,500 steps of the steepest descent method and 2,500 steps of the conjugate gradient method. Long-range Coulomb interactions were addressed using the particle mesh Ewald (PME) method (Darden et al., 1993). The SHAKE method was employed to constrain all the bonds connected to the hydrogen atom, allowing for an integration time step of 2 fs. For nonbonded interactions, the cutoff value was set at 10 Å. Under a constant volume, the entire system was heated from 0 to 300 K by using constraints for 60 ps Then the solvent density was equilibrated under a constant pressure system (T = 300 K, *p* = 1 atm). Finally, the sample was collected for 100 ns under constant pressure, and the frames were saved every picosecond for subsequent analysis.

Binding energy (ΔG_bind_) is the binding energy between the protein pocket and the ligand. It was calculated by the Molecular Mechanic/Poisson-Boltzmann Surface Area (MM/PBSA) method and according to the following equation: ΔG_bind_ = G_complex_−(G_protein_ + G_ligand_) ≈ ΔE_vdw_ + ΔE_ele_ + ΔG_sol_ where E_vdw_ and E_ele_ are the van der Waals and electrostatic energy and G_sol_ is the total solvation free energy (Miller et al., 2012; Li et al., 2021). The RMSD, RMSF values, and energy contribution for individual residues were imported into GraphPad Prism version 8.4.0 software to make the graph, respectively.

### Statistical Analysis

Statistical analysis was performed using GraphPad Prism version 8.4.0 software. The student’s t-test was used to analyze normal distribution data with homogeneity of variances. The Mann–Whitney U test was performed for nonparametric data. *p* values less than 0.05 were considered to be statistically significant.

## Results

### Virtual Screening for Potential METTL3 Inhibitors

To find some potential METTL3 inhibitors with high binding affinity in natural products, 1,042 natural products from commercially available sources were chosen to establish a screening library. The crystal structure of the METTL3-METTL14 complex (PDB code: 5IL1) was used to perform molecular docking (Wang et al., 2016). The ligand SAM involved in the cocrystallized METTL3-METTL14 complex was extracted and the compounds in the 1,042 natural products library were docked into the SAM binding site of METTL3 by AutoDock vina. After virtual screening, on the basis of the virtual screening scores (docking scores ≤ −7 kcal/mol) and readily available commercial sources, 14 compounds from the library were selected for further biological activity tests (Table 1).

**TABLE 1:** The molecular docking score of the selected 14 compounds after virtual screening and the ligands of METTL3 SAM.

NO.	Compound	CAS NO.	Formula	2D structural	Molecular docking score (kcal/mol) (*n* = 3)
1	Scutellarin	27,740-01-8	C_21_H_18_O_12_	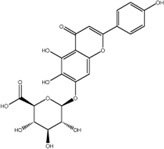	−10.3
2	Cynaroside	5,373-11-5	C_21_H_20_O_11_	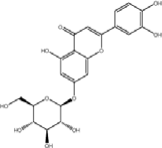	−9.73
3	Naringin	10,236-47–2	C_27_H_32_O_14_	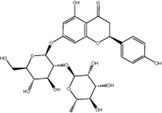	−9.43
4	Diosmetin	520-34-3	C_16_H_12_O_6_	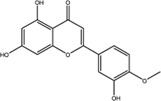	−9.43
5	Baicalein	491-67-8	C_15_H_10_O_5_	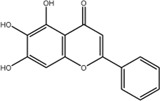	−9.4
6	Apigenin	520-36-5	C_15_H_10_O_5_	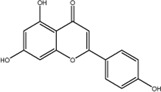	−9.2
7	Luteolin	491-70-3	C_15_H_10_O_6_	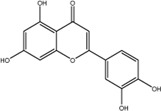	−9.17
8	Quercetin	117-39-5	C_15_H_10_O_7_	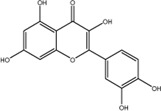	-9.1
9	Naringenin	480-41-1	C_15_H_12_O_5_	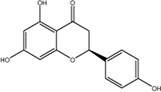	-8.97
10	Hesperitin	520-33-2	C_16_H_14_O_6_	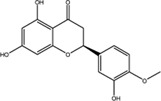	−8.83
11	Wogonin	632-85-9	C_16_H_12_O_5_	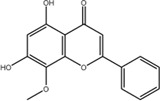	−8.5
12	Hesperidin	520-26-3	C_28_H_34_O_15_	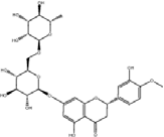	−7.73
13	Sophoricoside	152-95-4	C_21_H_20_O_10_	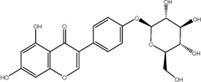	−7.63
14	Rutin	153-18-4	C_27_H_30_O_16_	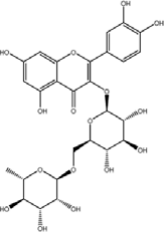	−7.2
-	SAM	29,908-03-0	C_15_H_22_N_6_O_5_S	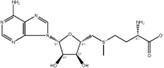	−7.37

### Initial Screening of Compounds by *In Vitro* Biological Activity Assay

**FIGURE 2:**
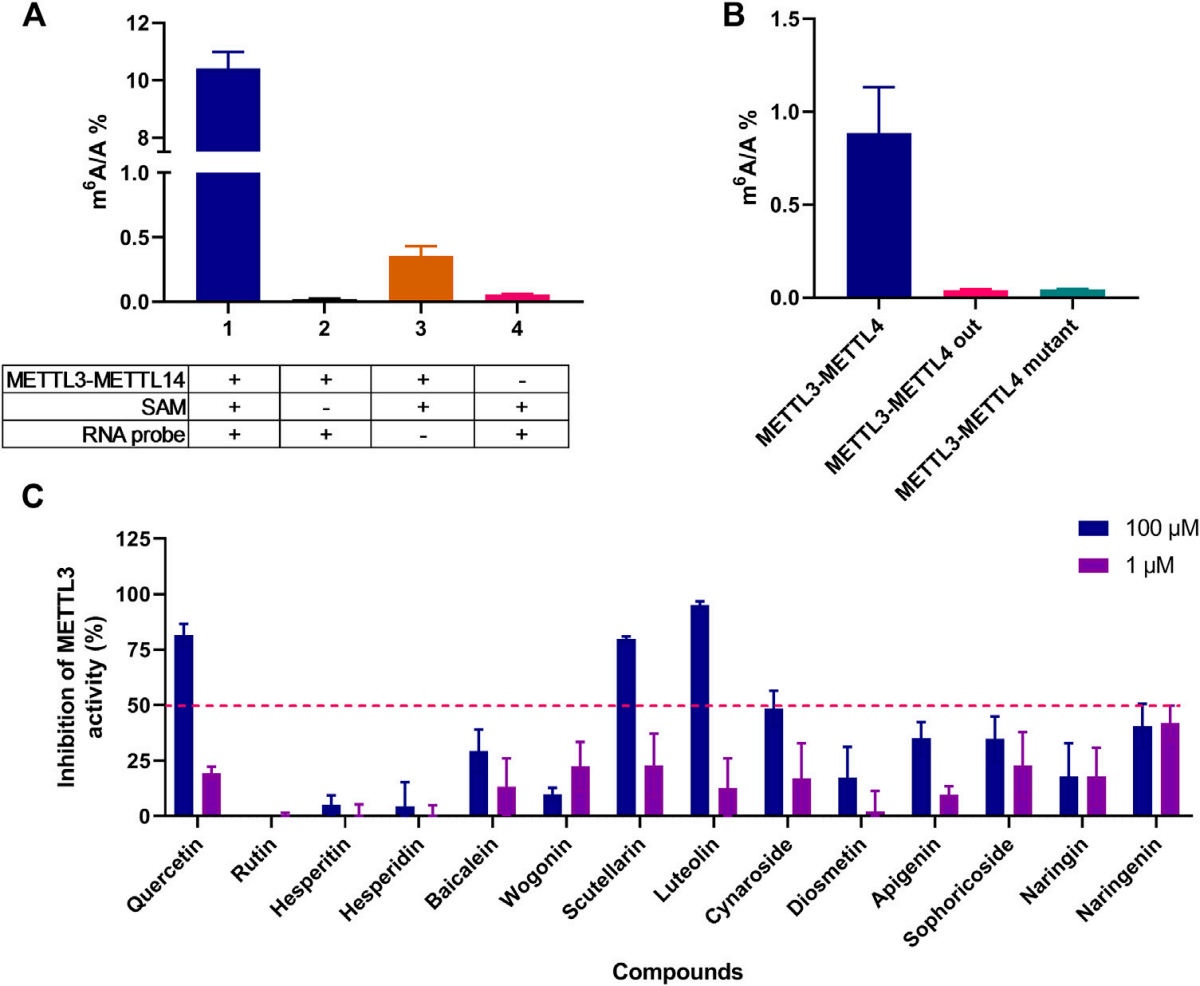
Initial screening of METTL3 inhibitors *in vitro*. **(A)** The influence of different components in the enzyme reaction system on the formation of m^6^A, group 1, 2, and 4, *n* = 4; group 3, *n* = 3. **(B)** METTL3-METTL14 mutant can hardly catalyze the formation of m^6^A, *n* = 4. **(C)** The inhibition rate was detected in the presence of 1 and 100 μM test compounds by using LC-MS/MS determination of the m^6^A/A ratio, *n* = 4. Data are mean ± SD.

In the METTL3-METTL14 activity assay, the inhibition of the m^6^A/A ratio was applied to evaluate the methyltransferase inhibitory activity and the LC-MS/MS method was used to determine the m^6^A content. As shown in Figures 2A, B, METTL3-METTL14 (wild type), SAM, and RNA are essential for m^6^A formation, and the METTL3-METTL14 mutant in the reaction mixture could hardly catalyze the formation of m^6^A; this finding confirmed the enzyme reaction specificity of this system. The 14 selected compounds (1 and 100 μM) were preliminary evaluated to identify their METTL3-METTL14 inhibitory activity. The initial screening compounds are shown in Figure 2C; the test compounds exhibited only slight or no METTL3 inhibitory activity in 1 μM concentration, while quercetin, scutellarin, and luteolin showed more than 50% inhibition at 100 μM; thus, these three compounds were chosen for further studies.

### Quercetin Dose-Dependently Inhibited METTL3 Activity

**FIGURE 3:**
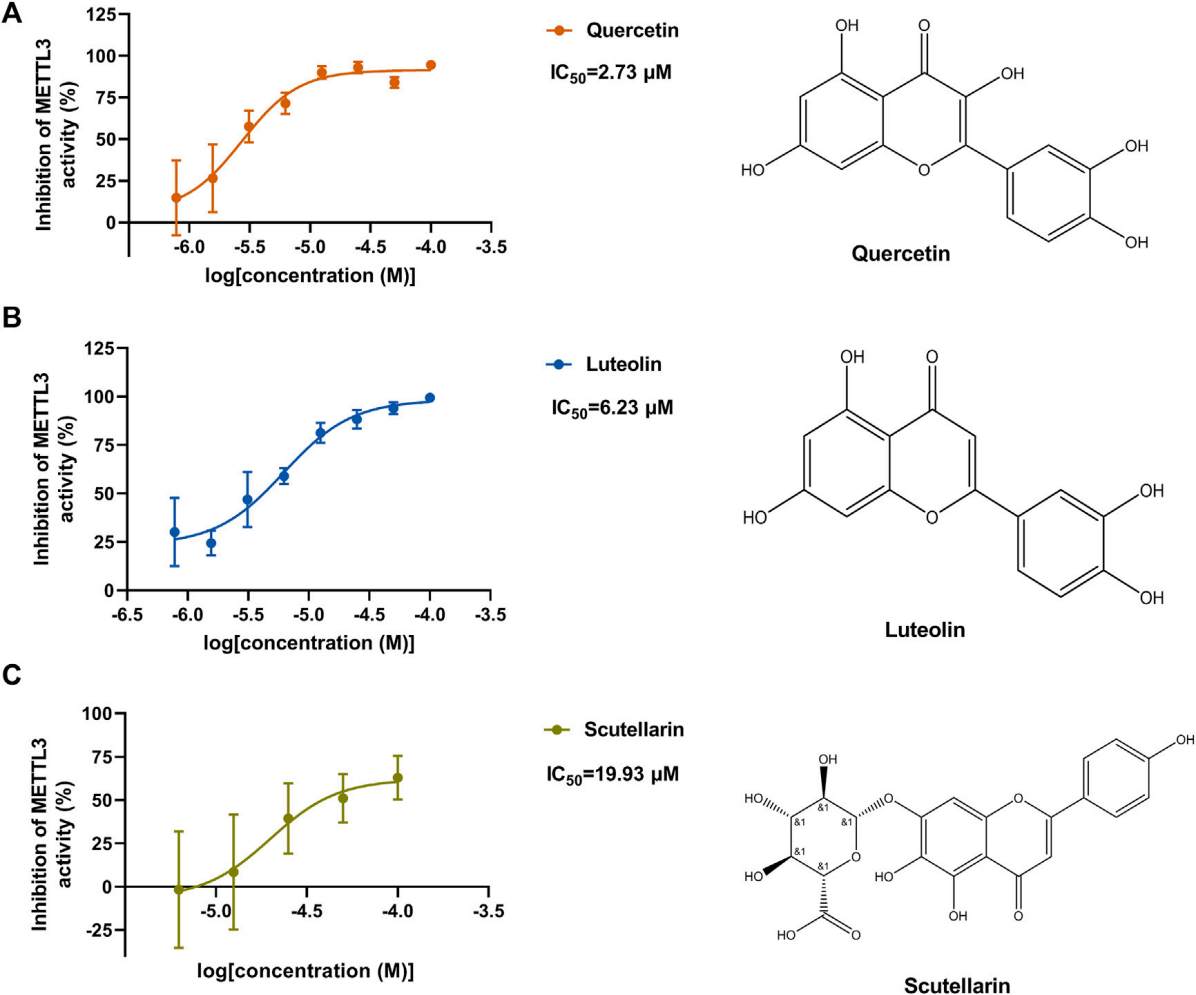
Dose-dependent response curves of the inhibition of the METTL3 enzyme by quercetin (n = 4) **(A)**, luteolin (*n* = 4) **(B)** and scutellarin (*n* = 3) **(C)**. The right side shows the molecular structure. Data are mean ± SD.

According to the results of the initial screening shown in Figure 2C, the dose-response analysis was performed for the three compounds, namely, quercetin, scutellarin, and luteolin. As shown in Figure 3, quercetin and luteolin at 0.78–100 μM concentration and scutellarin at 6.25–100 μM concentration inhibited METTL3 activity to varying degrees. The IC_50_ values of quercetin, luteolin, and scutellarin were 2.73, 6.23, and 19.93 μM, respectively. These results showed that quercetin is the most potent METTL3 methyltransferase activity inhibitor among the screened compounds, and it was selected for further research.

### Quercetin Inhibited m^6^A Methylation in Cells

**FIGURE 4:**
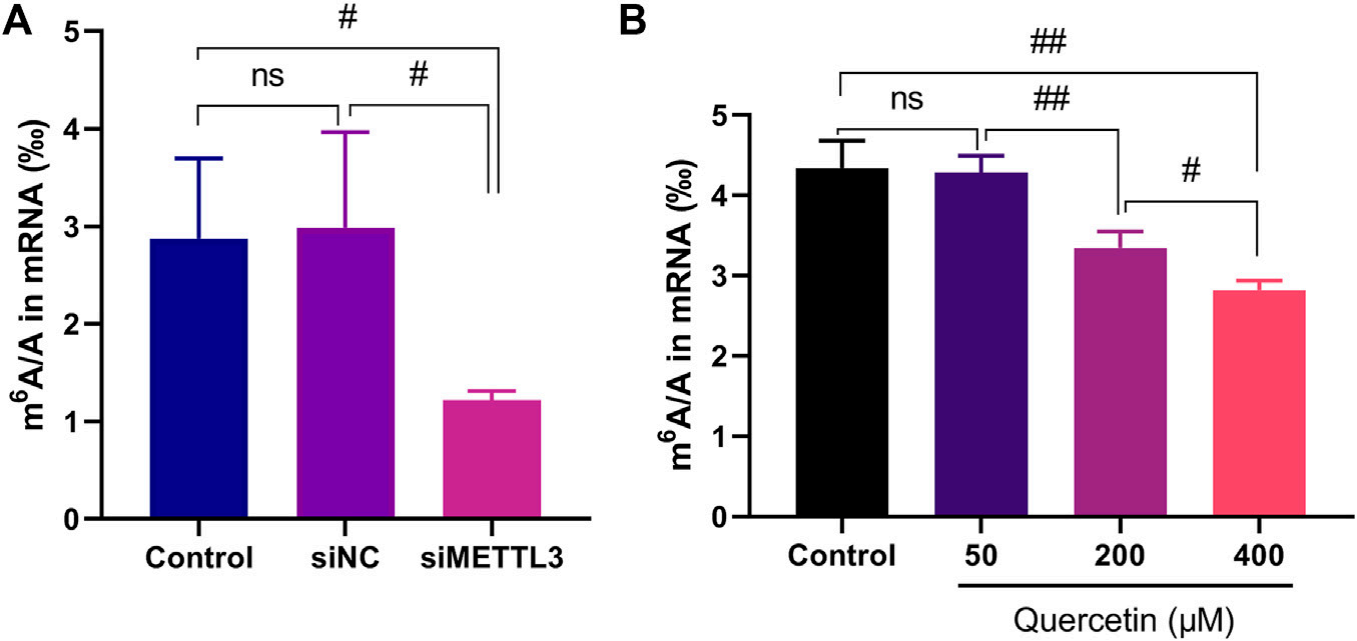
Cellular activity of siRNA mediated METTL3 knockdown and METTL3 inhibitor quercetin in MIA PaCa-2 cells. **(A)** Results of m^6^A content in mRNA from MIA PaCa-2 cells after transfection with siMETTL3, siNC or non-transfected cells (control), *n* = 4. **(B)** Results of m^6^A content in mRNA from MIA PaCa-2 cells after 24 h treatment with the indicated quercetin concentrations, *n* = 3. Data are mean ± SD. #, *p* < 0.05; ##, *p* < 0.01, ns, not significant.

Our study evaluated whether quercetin modulates m^6^A modification levels in cellular mRNA. First, the human pancreatic adenocarcinoma cell line MIA PaCa-2 was transiently transfected with siMETTL3 and siNC. Total RNA was extracted with TRIzol reagent, and mRNA was isolated by Oligo (dT) magnetic beads and digested into nucleosides. The m^6^A/A ratio in the cell was then determined by LC-MS/MS. The results showed that siRNA knockdown of METTL3 notably reduced the m^6^A level in the mRNA of MIA PaCa-2 cells (Figure 4A), indicating that METTL3 regulated m^6^A methylation modification in MIA PaCa-2 cells in a METTL3 activity-dependent manner; this finding is consistent with a previous report (Xia et al., 2019). The measured m^6^A methylation levels in MIA PaCa-2 cells ranged approximately from 1‰ to 4‰, which is similar to the reported human m^6^A abundance (Dominissini et al., 2012). After MIA PaCa-2 cells were treated with quercetin at 200 and 400 μM, the m^6^A level in the mRNA decreased significantly in a dose-dependent manner as compared to that in the untreated cells (DMSO alone) (Figure 4B). In addition, the level of METTL3 protein in MIA PaCa-2 cells treatment with quercetin at 200 and 400 μM was identified and the results showed that the m^6^A level reduction was not caused by a reduction in the METTL3 protein level (Supplementary Figure S1).

### METTL3 Inhibitor Quercetin Inhibited Tumor Cells Proliferation

**FIGURE 5:**
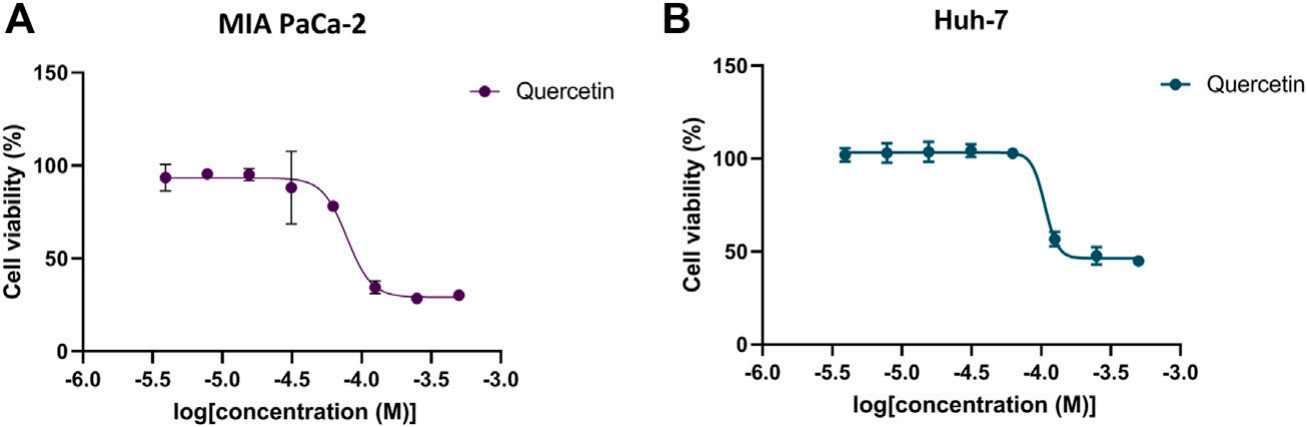
Inhibition of METTL3 affects the viability of two tumor cell lines. Dose-response curves of quercetin for of MIA PaCa-2 **(A)** and Huh7 cell lines **(B)**. Data are mean ± SD, *n* = 3.

To identify the effects of quercetin on tumor cell viability, a CCK-8 assay was used to evaluate the effect of quercetin on MIA PaCa-2 pancreatic cancer cells and Huh7 liver cancer cells. As shown in Figure 5, quercetin inhibited the viability of MIA PaCa-2 and Huh7 cells in a dose-dependent manner; this finding is consistent with previous reports (Harris et al., 2012; Brito et al., 2016). The IC_50_ values of quercetin for MIA PaCa-2 and Huh7 cells were 73.51 ± 11.22 μM and 99.97 ± 7.03 μM, respectively. The results demonstrated the METTL3 inhibitor quercetin decreased the proliferation of two different tumor cell lines, which is consistent with the effect of METTL3 knockdown. Accordingly, we speculated that the antitumor activity of quercetin might be related to the inhibition of METTL3 activity.

### Binding Mode Analysis

**FIGURE 6:**
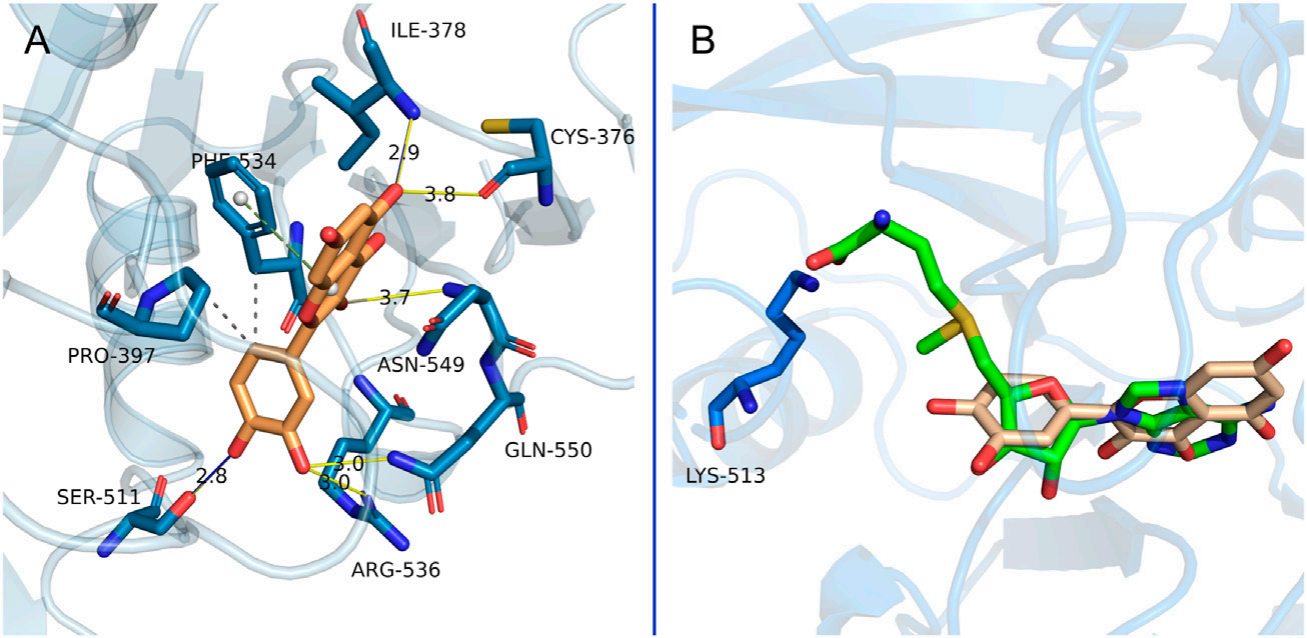
Putative interactions between METTL3-METTL14 and quercetin. **(A)** The main interactions between METTL3-METTL14 and quercetin are shown. **(B)** Overlay of the binding model of METTL3-METTL14 in complex with quercetin by docking and crystal structures of METTL3-METTL14 in complex with SAM (PDB code 5IL1). The carbon atoms in METTL3-METTL14 are shown in aqua blue. Carbon atoms in SAM and quercetin are shown in green and brown, respectively. The yellow or royal blue lines represent the hydrogen bonds. The gray dashed lines represent hydrophobic interactions. The green dashed line represents pi-stacking interaction.

The ligand-receptor interaction was determined by molecular docking to predict binding mode and affinity. As shown in Figure 6A, flavone C^3^-OH of quercetin forms a hydrogen bond with Asn549, C^5^-OH forms two hydrogen bonds with Ile378 and Cys376, C^3’^-OH forms two hydrogen bonds with Arg536 and Gln550, and C^4’^-OH forms a hydrogen bond with Ser511. On the other hand, the flavone C^6’^ group forms hydrophobic interactions with Pro397 and Phe534, while pyrone mediates further face-to-edge pi-stacking with Phe534. Either SAM or quercetin could bind to the target protein through hydrogen bond interactions (via Ile378, Arg536, Asn549, and Gln550) (Wang et al., 2016). Notably, the inhibitor quercetin fills the pocket of the adenosine moiety of SAM but not the pocket of the SAM methionine (Figure 6B), which probably provided selectivity against other SAM-dependent methyltransferases (Yankova et al., 2021).

### MD Simulation

**FIGURE 7:**
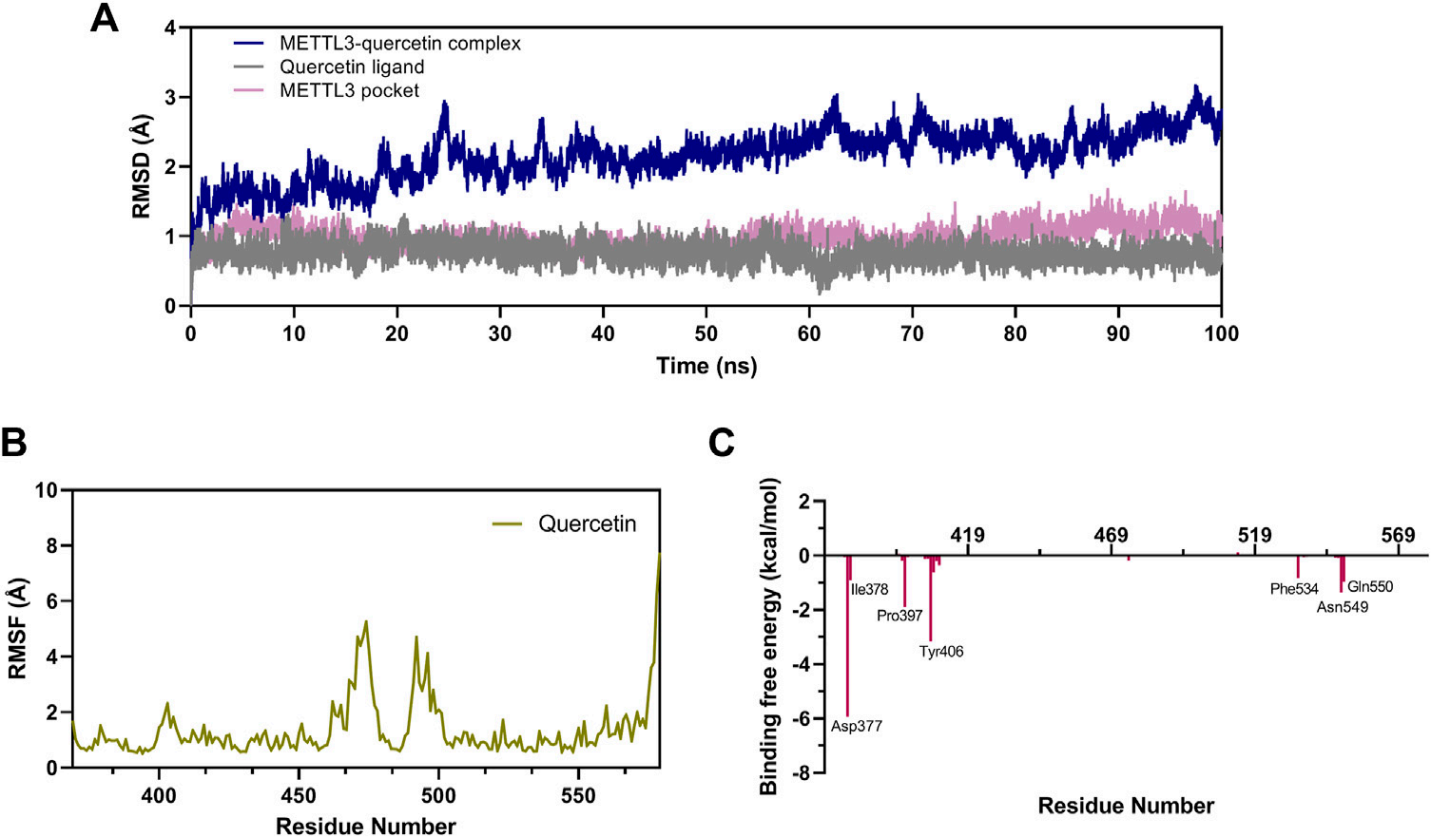
MD simulation studies in the METTL3-quercetin complex. **(A)** The RMSD of the backbone atoms of the METTL3-ligand systems for the METTL3 pocket, quercetin ligand and the METTL3-quercetin complex. **(B)** RMSF values of amino acid residues in the METTL3-quercetin system. **(C)** Energy contribution for individual residues 369–579 of the METTL3-quercetin complex.

MD simulation was performed to study the binding stability and dynamics of the quercetin-METTL3 complex at the atomistic level. A 100 ns MD simulation of the METTL3-quercetin system was performed using the sander program in Amber 20. The root mean square deviation (RMSD) of the backbone atoms in the METTL3-ligand system was calculated to explore the dynamic stability and conformational changes during the simulation. As shown in Figure 7A, the RMSD values of the METTL3-quercetin complex acquired equilibrium (within 25 ns) with very low fluctuations (below 1.5 Å), and the system remained stable up to 100 ns during the MD simulation, indicating that quercetin could bind to the METTL3 protein and stabilize the protein conformation rapidly. In addition, the RMSD values of the quercetin ligand and METTL3 pocket rapidly reached equilibrium within 2 ns.

To explore the binding stability of quercetin with METTL3, the root mean square fluctuation (RMSF) of the amino acids of METTL3 in the pocket was calculated to determine the flexibility of the protein backbone throughout the simulation. The RMSF plot of the METTL3-quercetin complex is shown in Figure 7B. The RMSF values of key common amino acid residues (such as Cys376, Ile378, Pro397, Ser511, and Phe534) were significantly lower than those in the other regions of METTL3. The amino acid fluctuations were also low in small molecule active pocket regions, which indicated that the state of residues was inflexible. These results revealed that these amino acid residues were strongly bound to quercetin, and the METTL3-quercetin complex was stable, which was consistent with the results of RMSD.

The MM/PBSA method was performed to calculate the binding affinities. The ΔG_bind_ for the METTL3-quercetin system was calculated to be −27.18 kcal/mol. As represented in Table 2, the electrostatic (ΔE_ele_) and van der Waals interactions (ΔE_vdw_) contributed to the major components of the ΔG_bind_ in this system, which indicated that electrostatic and van der Waals interactions played a key role in the interaction between METTL3 and quercetin. These results showed that quercetin exhibited a good binding affinity with METTL3 and the METTL3-quercetin complex is stable.

**TABLE 2:** Free energies of binding and intermolecular interaction energy components of the METTL3-quercetin complex obtained by the MM/PBSA approach (kcal/mol).

	Energy components	Energy (kcal/mol)
METTL3-quercetin	ΔE_vdw_	−30.44
	ΔE_ele_	−41.32
	ΔE_PB_	49.26
	ΔE_SA_	−4.68
	ΔG_sol_	44.58
	ΔG_bind_	−27.18

ΔG_PB_, and ΔG_SA_, are the electrostatic and nonpolar contributions to desolvation.

To identify the critical amino acid residues of METTL3 during binding with quercetin, the binding free energies were decomposed into individual residues by the MM/PBSA decomposition process. As shown in Figure 7C, the contributions of individual residues to the binding free energy varied in the range of +1.0 to −6 kcal/mol. Several residues (Asp377, Ile378, Pro397, Tyr406, Phe534, Asn549, and Gln550) are critical binding sites in the complex, most of which are consistent with the previous molecular docking results (Figure 6A). In conclusion, the analysis of RMSD, RMSF, and binding free energy confirmed that quercetin can bind efficiently to the pocket of the METTL3 protein and form a stable protein-ligand complex.

## Discussion

N6-Methyladenosine (m^6^A) is the most prevalent mRNA modification in mammalian cells, which is mainly catalyzed by the methyltransferase complex of METTL3-METTL14 (Wang et al., 2016). The dysregulation of m^6^A modification is associated with diseases such as cancer, drug resistance, and viral replication. Accumulating studies have demonstrated that METTL3 as a potential therapeutic target for cancers and virus infection (Hao et al., 2019; Zeng et al., 2020; Qiu et al., 2021; Yankova et al., 2021). For example, METTL3-mediated m^6^A mRNA methylation promoted the proliferation of hepatoblastoma through the regulation of CTNNB1 (Liu L. et al., 2019), while METTL3 promoted temozolomide resistance by increasing the expression of MGMT and ANPG (Shi et al., 2021). METTL3 also interacted with viral RNA-dependent RNA polymerase 3D and induced enhanced sumoylation and ubiquitination of the 3D polymerase that boosted viral replication (Hao et al., 2019). However, very limited research on the inhibitors of METTL3 has been reported. Despite being a huge drug resource, no natural products have been reported or screened as METTL3 inhibitors. The present study was performed to discover effective and safe METTL3 inhibitors from natural products for both biological and therapeutic purposes.

Recently, the computational technique of docking-based virtual screening has become an efficient method for rapidly discovering novel compounds from huge compound libraries (Grinter and Zou, 2014). In the present study, 1,042 natural products from commercially available sources were chosen to establish a screening library, and the molecular docking method by high-throughput docking into the SAM-binding site was then applied for high-throughput screening of METTL3 inhibitors from this library. A total of 14 compounds with docking scores ≤ −7 kcal/mol and readily available commercial sources were selected in the primary screening. To ensure the reliability of the experiment, biological tests were performed to detect activity after the virtual screening. An *in vitro* methyltransferase inhibition assay based on the determination of m^6^A content by LC-MS/MS was performed to screen and detect compounds inhibitory with activity for METTL3; this method is not only more intuitive and accurate to detect enzyme activity, but also has a lower running cost per sample. Three compounds with more than 50% inhibition at 100 μM concentration were then selected to detect their IC_50_ values for further evaluation. The most potent inhibitor, quercetin, showed an IC_50_ value of 2.73 μM in the METTL3 methyltransferase activity assay. To further investigate whether quercetin can modulate m^6^A modification levels in cellular mRNA, the level of m^6^A in the mRNA of the human pancreatic adenocarcinoma cell line was detected after treatment with quercetin or knockdown of METTL3. As expected, quercetin decreased the m^6^A modification level in cellular mRNA in a dose-dependent manner, while it did not affect the level of METTL3 protein. These findings demonstrated that the decrease of the m^6^A level in the mRNA of cells might be caused by the inhibition of METTL3 with the presence of quercetin inside cells. However, the cellular METTL3 specificity of quercetin and its potential use as a cellular METTL3 inhibitor will need more characterization in future research. Accumulating studies have demonstrated that m^6^A modifications are critical for most bioprocesses. METTL3 knockdown decreased RNA m^6^A modification and proliferation in pancreatic and liver cancer cells (Liu L. et al., 2019; Xia et al., 2019). Therefore, the effect of quercetin on the viability of tumor cells was evaluated. The results were consistent with the effect of METTL3 knockdown as reported earlier; quercetin inhibited the proliferation of four different tumor cell lines at the micromolar IC_50_ level (the IC_50_ values of PC9 and SKBR3 tumor cell lines were shown in Supplementary Figure S2). These findings provided evidence for METTL3 inhibitors as a promising strategy for anticancer therapy.

Since no structures of flavonoid or flavonol-type inhibitors in complex with METTL3 have been reported, molecular binding modeling was performed by molecular docking to understand the binding mode of quercetin with METTL3. Interestingly, the results indicated the inhibitor quercetin fills the pocket of the adenosine moiety of SAM but not the pocket of the SAM methionine, which probably provided selectivity against other SAM-dependent methyltransferases (Yankova et al., 2021). MD simulation and binding free energy analysis were performed to study the process and characteristics of quercetin binding with METTL3. The results revealed quercetin could bind efficiently to the pocket of the METTL3 protein and form a stable protein-ligand complex. In addition, the MM/PBSA decomposition process was carried out to analyze energy contribution for individual residues 369–579 of METTL3-quercetin complex. It was found that several residues (Asp377, Ile378, Pro397, Tyr406, Phe534, Asn549, and Gln550) have a critical binding free energy contribution, and most of them are consistent with the previous molecular docking results. These residues are predicted as energetically important for ligand binding inside the ligand binding site via hydrophobic, hydrogen bond, or pi-Stacking interactions in complexes. Overall, these results highlighted quercetin as a new METTL3 inhibitor.

Quercetin is a flavonol-type compound, which is one of the most abundant naturally occurring polyphenols in our foods (Andres et al., 2018). Our present study is the first to demonstrate that quercetin could inhibit METTL3 and decrease the m^6^A/A ratio in MIA PaCa-2 cells, which provides a base for targeted treatment of diseases, especially cancer. Although some potent inhibitors of METTL3 such as UZH1a and STM2457 (Moroz-Omori et al., 2021; Yankova et al., 2021) have been found recently, their adverse reactions and drug safety remain unknown. Quercetin, with a well-known pharmacokinetics and safety profile, can not only be considered a good candidate for further optimization and development but also may be tested in future clinical trials to enrich the drug arsenal (Abian et al., 2020; Russo et al., 2020). Previous studies have reported that the mechanisms of the anti-tumor activity of quercetin include the modulation of the PI3K/Akt/mTOR, Wnt/-catenin, and MAPK/ERK1/2 pathways (Reyes-Farias and Carrasco-Pozo, 2019). Yet, there are relatively few studies on its targets. Here we reported the target of quercetin and revealed its antitumor activity by detecting the effect on cell viability. In addition, the effects of quercetin in existing literature reports have a lot in common with blocking METTL3, such as antitumor effect, reversal of tumor resistance, and antiviral effect (Chen et al., 2010; Pan et al., 2020; Tang et al., 2020; Bastaminejad and Bakhtiyari, 2021). It is of interest to study whether this is related to quercetin-mediated METTL3 inhibition. In the current study, we proved quercetin could inhibit METTL3 activity and decreased the m^6^A level, while it did not affect the m^5^C/C level (Supplementary Figure S3). However, further study was needed to demonstrate the METTL3 specificity of quercetin and whether it has an effect on the expression level of other RNA modifications. Furthermore, the potential use of quercetin as METTL3 inhibitor and *in vivo* efficacy need to be studied to develop METTL3 inhibitor and further explore its potential antitumor activity. In future research, the structure of quercetin could be modified and optimized to improve its activity.

In conclusion, this study identified quercetin as the first METTL3 inhibitor derived from natural products, which could facilitate the development of METTL3-targeting drugs for the treatment of diseases. Moreover, this strategy offers a feasible and effective approach to discover novel potential METTL3 inhibitors.

## Data Availability

The original contributions presented in the study are included in the article/Supplementary Material, further inquiries can be directed to the corresponding author.

## Abbreviations

ATCC, American Type Culture Collection; CCK-8, Cell Counting Kit-8; E_ele_, electrostatic energy; E_vdw_, van der Waals energy; GAFF, general Amber force field; G_sol_, total solvation free energy; IC_50_, half-maximal inhibitory concentration; m^6^A, N^6^-methyladenosine; MD, molecular dynamics; METTL14, methyltransferase-like 14; METTL3, methyltransferase-like 3; PDB, Protein Data Bank; PME, the particle mesh Ewald; RESP, restrained electrostatic potential; RMSD, the root mean square deviation; RMSF, the root mean square fluctuations; SAH, S-adenosyl homocysteine; SAM, S-adenosyl-L-methionine; siNC, nonspecific siRNA oligonucleotides; WTAP, Wilms’ tumor 1-associating protein; ΔG_bind_, binding energy.
