# Supplementary material for: Discovery of METTL3 Small Molecule Inhibitors by Virtual Screening of Natural Products
Source: Front Pharmacol. 2022 Apr 27;13:878135. doi: 10.3389/fphar.2022.878135 (PMC9093036; doi:10.3389/fphar.2022.878135)
Supplement: Supplementary file 1 [file DataSheet1.docx]

Supplementary Material

## Supplementary Figures


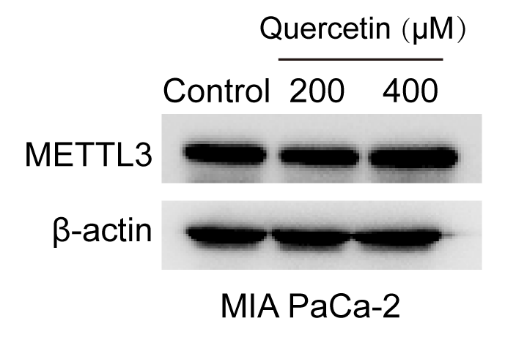


**Supplementary Figure S1.** Western blotting of METTL3 expression level in MIA PaCa-2 cells after treatment with 200 and 400 μM of quercetin for 24 h. The following antibodies were used: anti-human METTL3 (1:1000 dilution, Abcam, #ab195352), anti-β-actin (1:5000 dilution, Proteintech, #66009-l-lg), goat anti-mouse IgG (1:7500 dilution, Jackson,#115-035-003) and goat anti-rabbit IgG (1:7500 dilution, Jackson, #111-035-003).


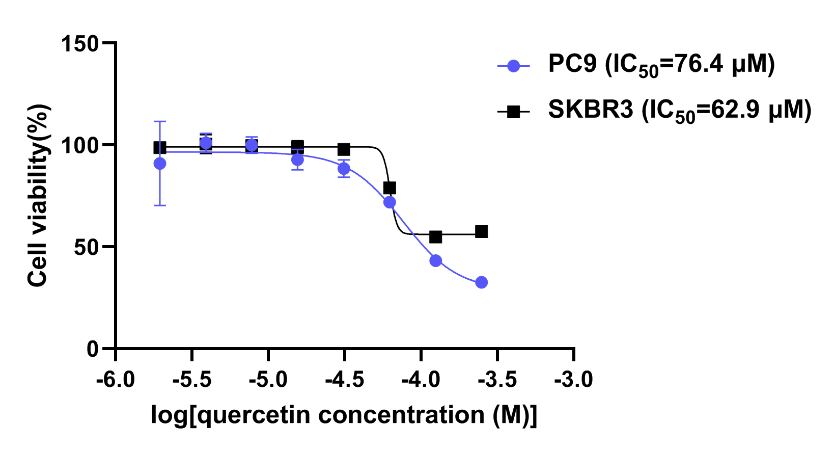


**Supplementary Figure S2.** Quercetin dose-response curves for PC9 and SKBR3 cell lines. Data are mean ± SD, n = 6. IC_50_ for each cell line is shown in brackets.


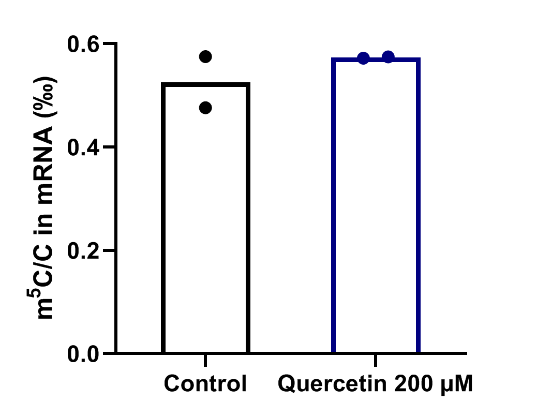


**Supplementary Figure S3.** Results of m^5^C content in mRNA from MIA PaCa-2 cells after 24h treatment with 200 μM quercetin concentrations. The m^5^C/C ratio was determined by LC-MS/MS. Quercetin did not affect the MIA PaCa-2 cells m^5^C level.
